# Identification of vitamin D-related signature for predicting the clinical outcome and immunotherapy response in hepatocellular carcinoma

**DOI:** 10.1097/MD.0000000000037998

**Published:** 2024-05-10

**Authors:** Tianyi Wang, Lulu Han, Jinjiang Xu, Bin Guo

**Affiliations:** a Department of Physical Examination, the First Affiliated Hospital of Jinzhou Medical University, Jinzhou, Liaoning, China; b Department of Physical Examination, Jinzhou Medical University, Jinzhou, Liaoning, China.

**Keywords:** HCC, Immunotherapy, machine learning, Prognostic signature, Vitamin D

## Abstract

Hepatocellular carcinoma (HCC) is one of the most common cancers globally, seriously endangering people health. Vitamin D was significantly associated with tumor progression and patients’ prognosis. Integrative 10 machine learning algorithms were used to develop a Vitamin D-related signature (VRS) with one training cohort and 3 testing cohorts. The performance of VRS in predicting the immunology response was verified using several predicting approaches. The optimal VRS was constructed by stepCox + superPC algorithm. VRS acted as a risk factor for HCC patients. HCC patients with high-risk score had a poor clinical outcome and the AUCs of 1-, 3-, and 5-year ROC were 0.786, 0.755, and 0.786, respectively. A higher level of CD8 + cytotoxic T cells and B cells was obtained in HCC patients with low-risk score. There is higher PD1&CTLA4 immunophenoscore and TMB score in low-risk score in HCC patients. Lower TIDE score and tumor escape score was found in HCC cases with low-risk score. The IC50 value of camptothecin, docetaxel, crizotinib, dasatinib, and erlotinib was lower in HCC cases with high-risk score. HCC patients with high-risk score had a higher score of cancer-related hallmarks, including angiogenesis, glycolysis, and NOTCH signaling. Our study proposed a novel VRS for HCC, which served as an indicator for predicting clinical outcome and immunotherapy responses in HCC.

## 1. Introduction

Hepatocellular carcinoma (HCC) is one of the most common cancers globally, seriously endangering people health.^[1]^ There are over 900,000 new diagnosed HCC cases and over 830,000 HCC-related deaths around the world in 2022.^[2]^ Many HCC patients lose the opportunity for surgery, because they are usually diagnosed at advanced stages.^[3]^ Less than 1/5 of HCC patients survive more than 5 years.^[3]^ High heterogeneity, invasiveness and metastasis are the main reasons leading to the poor clinical outcome of HCC patients.^[4]^ Limited biomarkers could be used for predicting drug sensitivity and clinical outcome clinically.

Vitamin D is a fat-soluble vitamin,^[5]^ which could be obtained from diet or from sunlight-dependent endogenous synthesis of the lower epidermis.^[6]^ Study showed that vitamin D could help control tumors and prolong the lives of cancer cases.^[7]^ Moreover, lack of vitamin D is correlated with higher cancer incidence rate.^[8]^ Vitamin D suppressed tumor growth and involved in the development of cancer.^[9]^ Leyssens et al found that vitamin D was involved in the proliferation and differentiation in CRC.^[10]^ Vitamin D metabolism-related gene CYP24A1 was overexpressed in some types of cancer and correlated with tumor growth.^[11,12]^ Considering the vital role of vitamin D in cancer, it is necessary to explore the role of vitamin D-related genes in HCC.

Based on bulk RNA-seq data, our study developed a vitamin D-related signature (VRS) for HCC cases. The correlation between VRS and drug sensitivity of HCC patients was evaluated by several predicting scores.

## 2. Materials and methods

### 2.1. Data acquisition

Bulk RNA-seq datasets of HCC were obtained from the Cancer Genome Atlas (TCGA) (n = 329), ICGC (n = 228), GSE14520 (n = 218) and GSE72094 (n = 90) datasets. Metastatic HCC should be excluded from our study. IMvigor210 (n = 298) and GSE91061 (n = 89) dataset were utilized to explore the linked between the VRS and immunotherapy response. A total of 194 vitamin D-related gene lists were obtained from 2 literature pieces.^[5,13]^ Among them, 173 vitamin D-related genes were identified from TCGA.

### 2.2. Integrative machine learning algorithms constructed an optimal VRS

DEGs were identified by “limma” package with the cutoff of |LogFC| ≥ 1.5. Risk factors were identified by univariate Cox analysis. An integrative analysis procedure with 10 machine learning algorithms was used to develop a predicting VRS for HCC. The candidate genes in VRS and corresponding coefficient were firstly obtained in TCGA cohort. The VRS score and C-index of HCC cases were then calculated in all cohorts. The prognostic signature with the highest average C-index was regarded as the optimal VRS.

### 2.3. Assessment of VRS

Based on the best cutoff determining by the survminer R package, HCC cases were separated into groups. We then drew ROC curve and C-index using the “survival” R package. The potential prognostic biomarkers were obtained after performing Cox analyses (Univariate and multivariate). The construction of predictive nomogram was determined by “nomogramEx” package using VRS score and other clinical parameters. To evaluate the possibility of VRS for clinical application, we then draw a decision curve analysis (DCA) curve.

### 2.4. Immune infiltration analysis

ESTIMATE analysis was performed to calculate ESTIMATEScore of HCC patients.^[14]^ Immunedeconv (integrating 6 state-of-the-art algorithms) was utilized to explore the correlation between VRS score and immune cell.^[15]^ The level of immune cells, immune-related activities or functions scores were analyzed with ssGSEA method. The GSVA package was used to calculate the score of the gene set of “h.all.v7.4.symbols.gmt.”

### 2.5. Drug sensitivity analysis

Several predicting scores (TMB score, immunophenoscore, tumor escape score and TIDE score) were utilized to explore the value of VRS in immunotherapy benefit predicting of HCC cases. There is less likelihood of immune escape and better immunotherapy benefit in patients with lower TIDE score and higher TMB score. Bu using the “oncoPredict” R package, we then obtained The IC50 of drugs in each HCC case

## 3. Results

### 3.1. Integrative machine learning algorithms developed an optimal prognostic VRS

**Figure 1.**
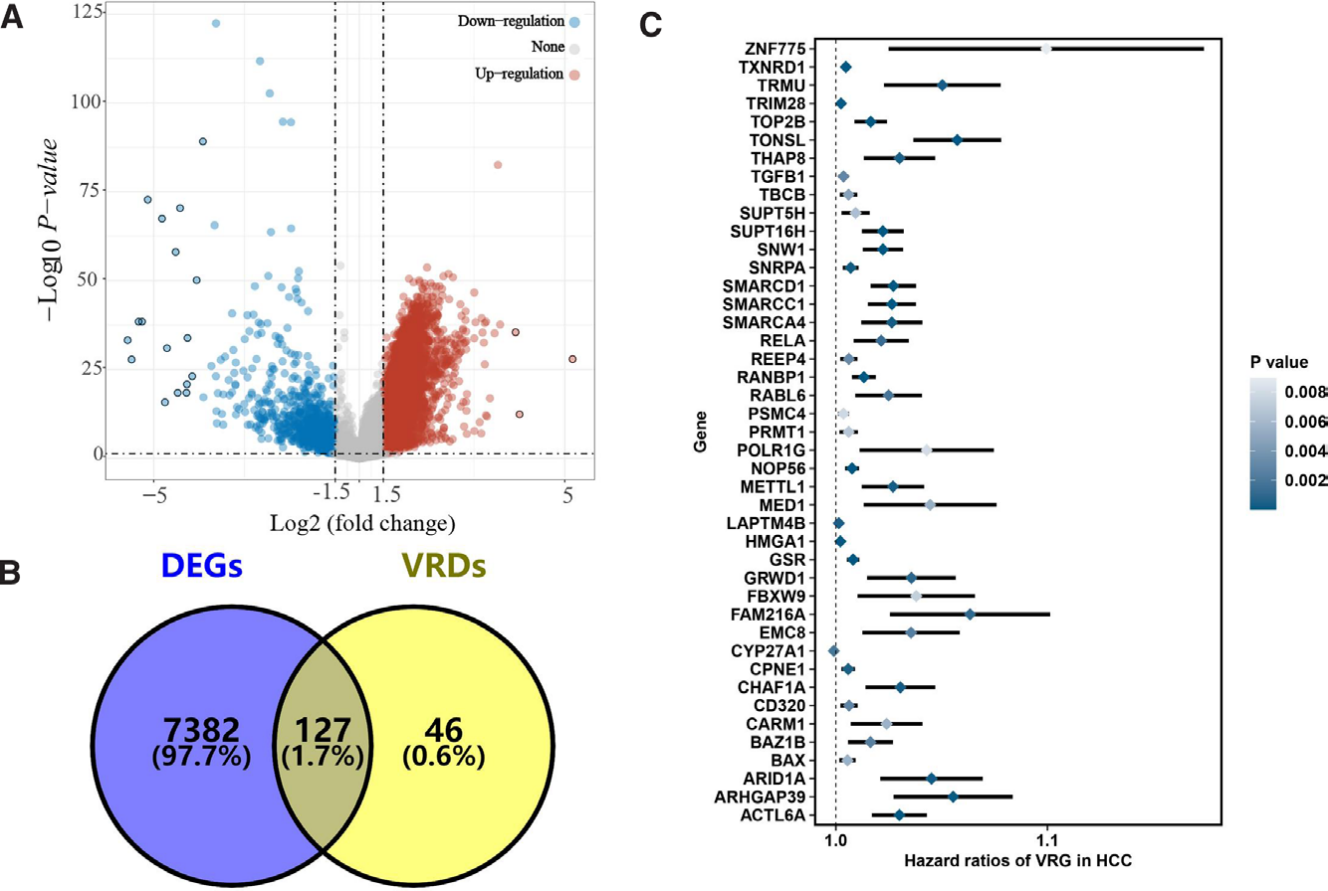
Identification of potential prognostic biomarkers of vitamin D-related genes in HCC. (A) Heatmap showing the differentially expressed genes in HCC. (B) The overlap of vitamin D-related genes and differentially expressed genes in HCC. (C) The potential biomarkers of vitamin D-related genes in HCC. HCC = hepatocellular carcinoma.

**Figure 2.**
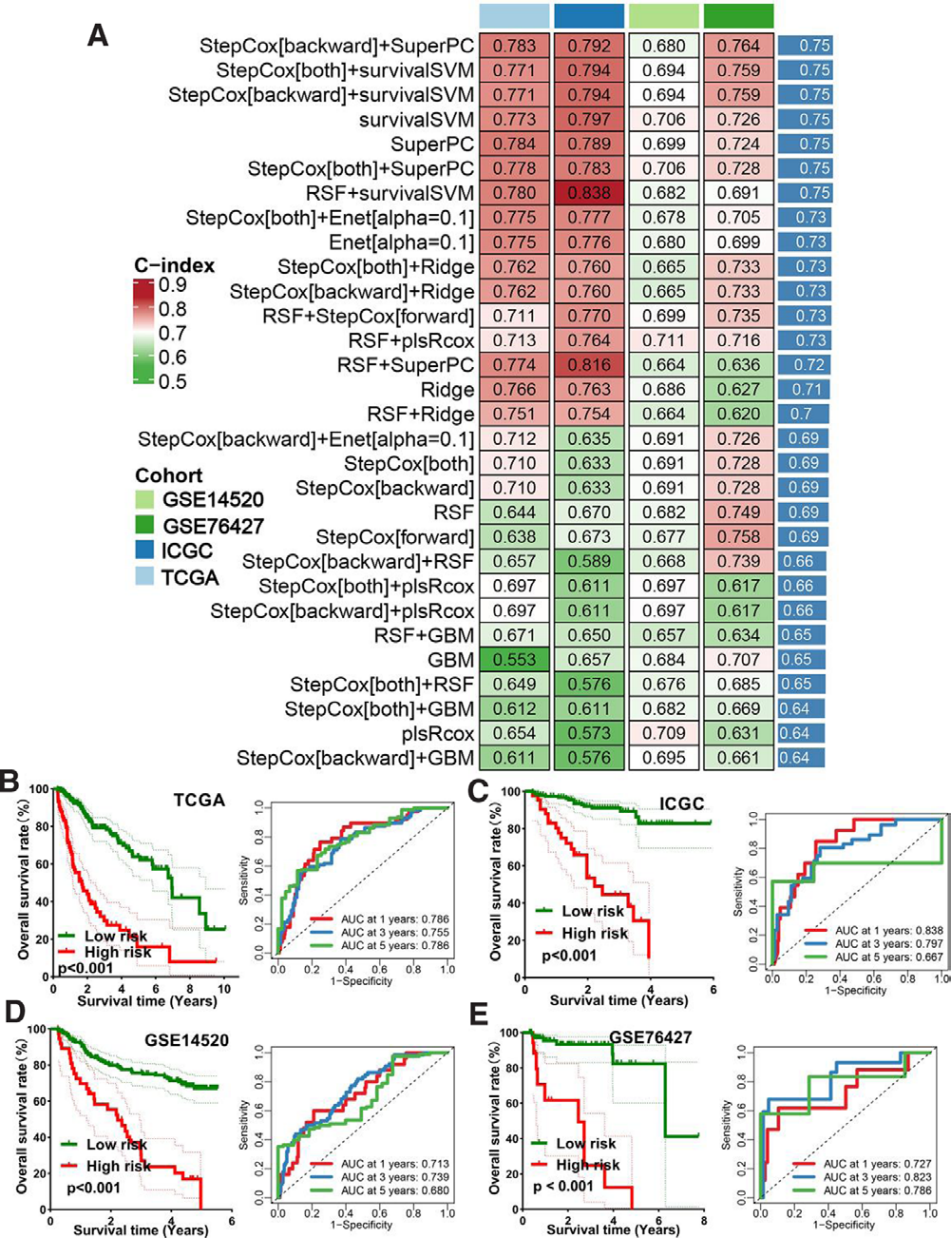
Identification and evaluation of a Vitamin D-related prognostic signature (VRS). (A) The C-index of each prognostic model constructed by 10 machine learning algorithms in training and testing cohort. The survival curve and corresponding ROC curve of HCC with high and low-risk score in TCGA (B), ICGC (C), GSE14520 (D) and GSE76427 (E) cohort. HCC = hepatocellular carcinoma, TCGA = the Cancer Genome Atlas.

A total of 7509 DEGs were obtained in HCC with |LogFC| ≥ 1.5 as the threshold (Fig. 1A). Among these DEGs, a total of 127 were differentially expressed VRGs (Fig. 1B). Cox univariate analysis suggested that 43 differentially expressed VRGs were significantly correlated with the prognosis of HCC patients (Fig. 1C, *P* < .05). We obtained 30 kinds of prognostic models with 10 machine learning-based methods and the C-index of each prognostic model was shown in Figure 2A. As a result, the model constructed by stepCox[backward] + SuperPC method was suggested as the optimal model with a highest average C-index of 0.75 (Fig. 2A). The VRS was constructed by 12 VRGs, and the risk score (VRS score) of each HCC case was calculated with the formula: risk score = 0.553 × CYP24A1^exp^ + 0.123 × NCOA7^exp^ + 0.459 × TGFB1^exp^ + (−0.389) × GSR^exp^ + 0.345 × IGFBP3^exp^ + 0.463 × IGFBP2^exp^ + (−1.053) × VGF^exp^ + (−0.362) × AGAP2^exp^ + 0.264 × DENND6B ^exp^ + 0.314 × LRRC8A^exp^ + (−0.332) × BCL6^exp^ + 0.423 × FCER2^exp^. With the best cutoff, HCC cases were separated into high and low-risk groups. A poor OS rate was observed in HCC patients with high-risk score in TCGA (*P* < .001), ICGC (*P* < .001), GSE14520 (*P* < .001), and GSE76427 (*P* < .001) cohort, with 1-, 3-, and 5-year AUCs of 0. 766, 0.755, and 0.786 in TCGA cohort; 0.838, 0.797, and 0.667 in ICGC cohort; 0.713, 0.739, and 0.680 in GSE14520 cohort; and 0.727, 0.823, and 0.786 in GSE76427 cohort, respectively (Fig. 2B–E).

### 3.2. Evaluation of the performance of VRS

**Figure 3.**
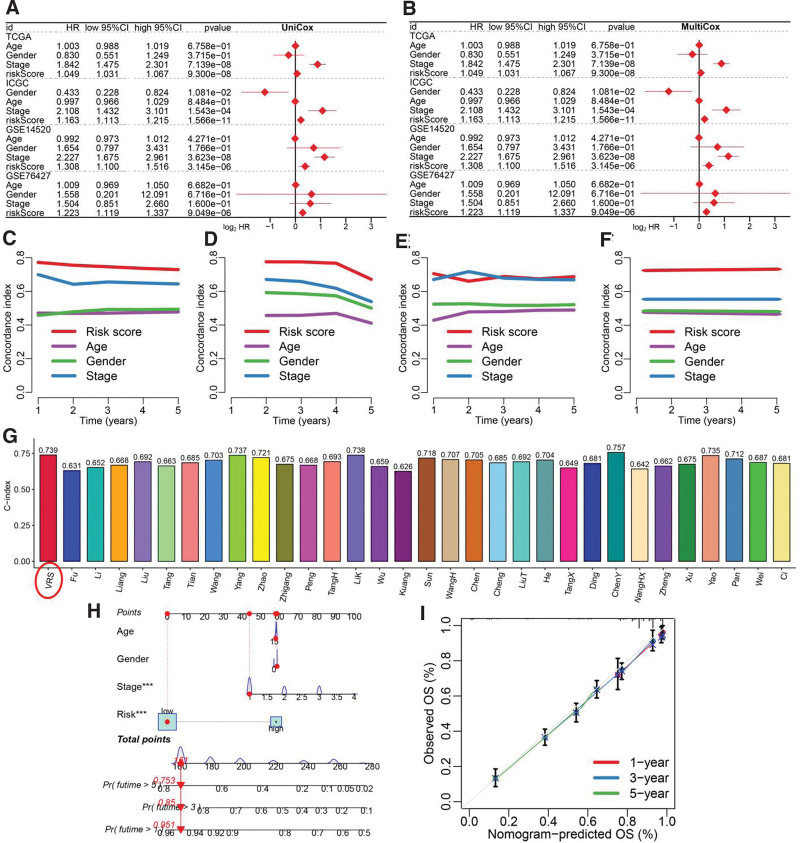
Evaluation of the performance of Vitamin D-related signature (VRS). Univariate (A) and multivariate (B) Cox regression analysis identified risk factor for HCC. (C–F) C-index curve evaluated the discrimination of VRS in predicting the overall survival rate of HCC patients in training and testing cohort. (G) The C-index of risk models had been developed for HCC. (H) A predictive nomogram constructing with risk score, age, gender and clinical stage. (I) Calibration plots demonstrated that the actual 1-yr, 3-yr, and 5-yr survival times were highly consistent with the predicted survival times. HCC = hepatocellular carcinoma.

Univariate and multivariate Cox regression analysis suggested VRS-based risk score as an independent risk factor for the clinical outcome of HCC patients in TCGA, ICGC, GSE14520 and GSE76427 cohort (Fig. 3A–B). The C-index of VRS-based risk score was higher than that of age, gender and clinical stage (Fig. 3C–F). We also randomly collected 31 prognostic signatures that have constructed for HCC (Supplementary Table 1, http://links.lww.com/MD/M326) and calculated their C-index. As a result, the C-index of VRS was higher than that most of these 31 prognostic signatures, excepted ChenY signature (Fig. 3G). To further evaluate the mortality risk at 1, 3 and 5 years of HCC patients, we then constructed a predictive nomogram (Fig. 3H). The calibration curves demonstrated the relative well conformity between speculated outcomes and observed outcomes (Fig. 3I).

### 3.3. VRS-based distinct ecosystem in HCC

**Figure 4.**
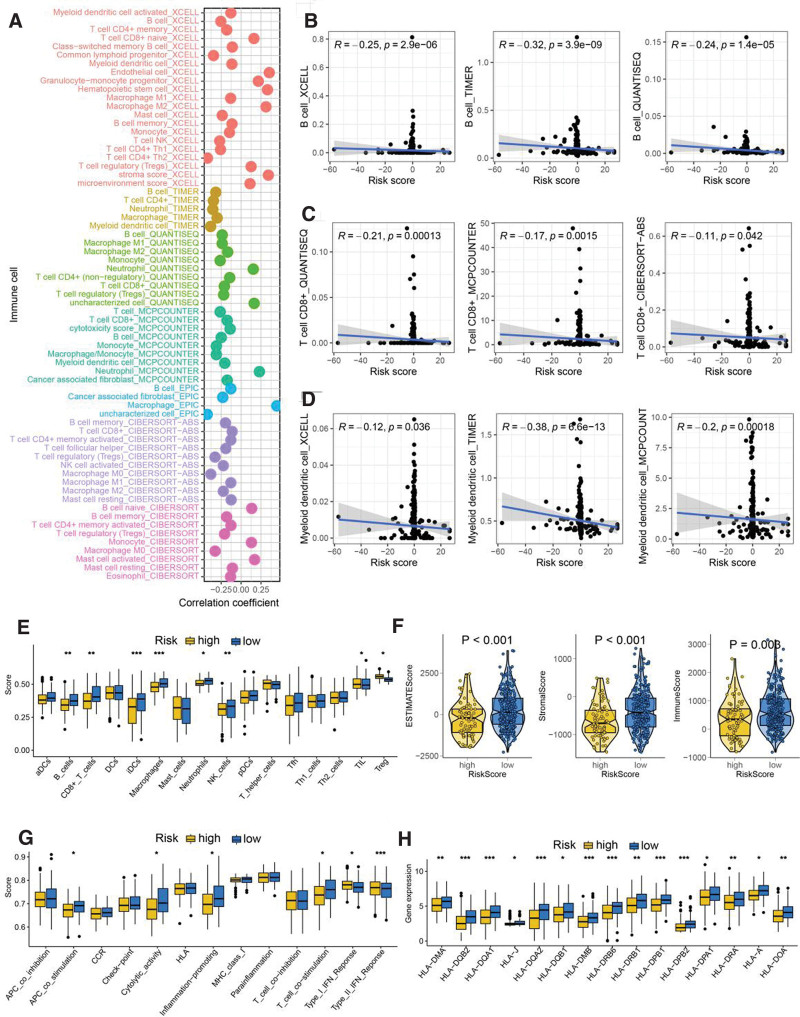
Immune microenvironment landscape in HCC patients with different risk score. (A) The correlation between risk score and immune cell infiltration based on several state-of-the-art algorithms. Risk score showed negative correlation with the abundance of B cells (B), CD8 + T cells (C) and dendritic cells (D). (E) The level of immune cells in HCC patients with different risk score based on ssGSEA analysis. (F) Low-risk score indicated a higher stromal score, immune score, and ESTIMAE score. (G–H) The score of immune-related functions and HLA-related genes in HCC patients with different risk scores. *P < .05, **P < .01, ***P < .001. HCC = hepatocellular carcinoma.

Significant correlation was obtained between risk score and immune cells (Fig. 4A), including B cells, CD8 + T cells and dendritic cells (Fig. 4B–D). Moreover, a higher abundance of immunoactivated cells was obtained in HCC patients with low-risk score, including B cells, CD8 + T cells and NK cells (Fig. 4E). HCC patients with low-risk score had a higher stromal score, immune score and ESTIMAE score (Fig. 4F, all *P* < .05). We also found a higher cytolytic score, inflammation-promoting score and T cell co-stimulation score in HCC patients with low-risk score (Fig. 4G). Further analysis revealed that the level of most of the HLA-related genes was higher in HCC patients with low-risk group (Fig. 4H).

### 3.4. VRS acted as an indicator for the drug sensitivity in HCC

**Figure 5.**
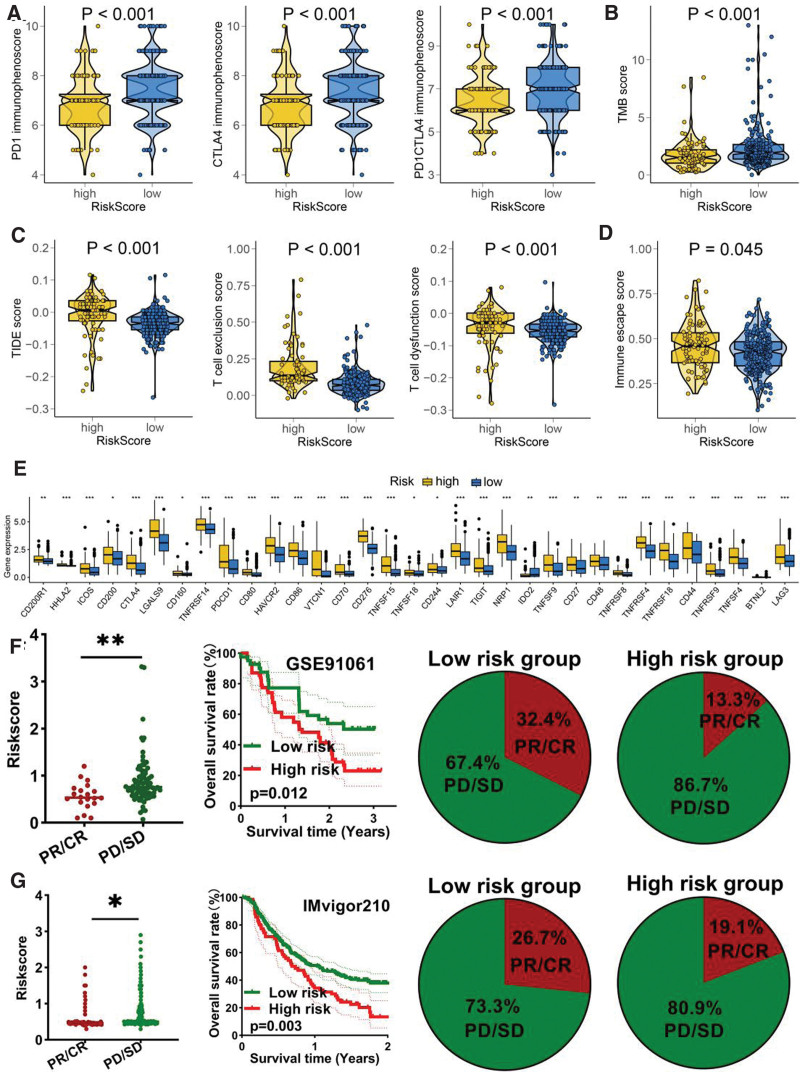
Vitamin D-related signature as an indicator for immunotherapy response in HCC. (A–B) The immunophenoscore and TMB score in HCC patients with high- and low-risk score. (C–D) The TIDE, T cell dysfunction score, T cell exclusion score and immune escape score in HCC patients with high- and low-risk score. (E) The level of common immune checkpoints in HCC patients with high- and low-risk score. The overall rate and immunotherapy response rate in patients with high- and low-risk score in GSE91061 (F) and IMvigor210 (G) cohort. *P < .05, **P < .01, ***P < .001. HCC = hepatocellular carcinoma.

**Figure 6.**
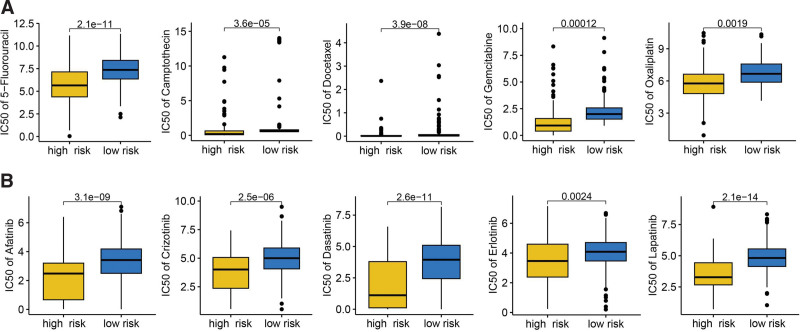
The IC50 value of common drugs for chemotherapy and targeted therapy. High-risk score indicated a lower IC50 value of common drugs for chemotherapy (A) and target therapy (B) in HCC patients with high-risk score. HCC = hepatocellular carcinoma.

Several approaches were used to evaluate the predictive value of VRS-based risk score in immunotherapy response. The result suggested higher PD1 immunophenoscore, CTLA4 immunophenoscore, and PD1&CTLA4 PD1 immunophenoscore in HCC with low-risk score (Fig. 5A, *P* < .05). Compared with high-risk score group, low-risk score group had a higher TMB score in HCC patients (Fig. 5B, *P* < .001). Moreover, HCC patients with low-risk score had a lower TIDE score, T cell dysfunction score, T cell exclusion score (Fig. 5C, all *P* < .05). A lower immune escape score was obtained in HCC patients with low-risk (Fig. 5D, *P* = .045). Immune checkpoints played a vital role in inhibiting immune responses and promoted self-tolerance in cancer. In our study, we found that the level of most of immune checkpoints was lower in HCC patients with low-risk score (Fig. 5E, all *P* < .05). This evidence showed that HCC with low-risk score may have a better sensitivity to immunotherapy. We also verified our results using 2 immunotherapy cohort. In GSE91061 cohort, the risk score in non-responders was higher and patients with high-risk score had a poor OS rate (Fig. 5F, *P* < .05). Moreover, the response rate in high-risk score group was significant lower (Fig. 5F). Similar results were obtained in IMvigor210 cohort (Fig. 5G). We also explored the IC50 value of common drugs for chemotherapy and targeted therapy in HCC. The result suggested a lower IC50 value of chemotherapy drug (5-fluorouracil, camptothecin, docetaxel, gemcitabine, Oxaliplatin, and paclitaxel) and targeted therapy drug (afatinib, crizotinib, dasatinib, erlotinib, and lapatinib) in HCC patients with high-risk score (Fig. 6A–B). Thus, HCC with high-risk score may be better sensitivity to chemotherapy and targeted therapy.

### 3.5. The distinct difference in cancer-related hallmarks in different VRS-based risk score group

**Figure 7.**
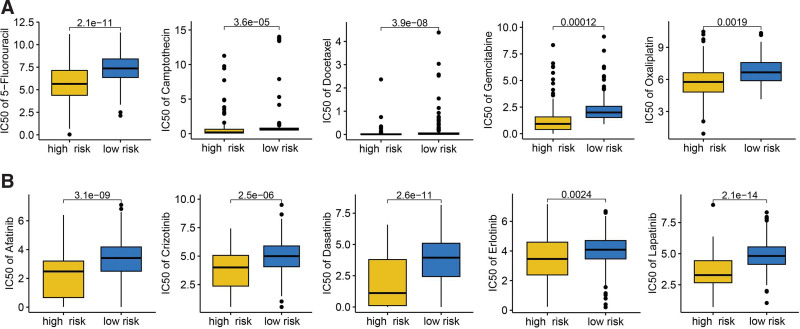
Gene set enrichment analysis of Vitamin D-related prognostic signature.

We then performed gene set enrichment analysis to explore the biological process in HCC patients with different VRS score. As shown in Figure 7, HCC patients with high-risk score had a higher score of angiogenesis, coagulation, DNA repair, glycolysis, hypoxia, IL2_STAT5 signaling, MTORC1 signaling, and P53 pathway, indicating that the activation of these biological processes may play the vital role in HCC tumorigenesis and progression.

## 4. Discussion

In our study, a total of 10 integrative machine learning algorithms was used to construct a VRS, which was verified by using 3 validation cohort. The optimal VRS was constructed by stepCox + superPC algorithm. VRS acted as a risk factor for HCC patients. HCC patients with high-risk score had a poor clinical outcome and the AUCs of 1-, 3-, and 5-year ROC were 0.786, 0.755, and 0.786, respectively. Moreover, we also found that VRS acted as an indicator for predicting drug sensitivity in HCC.

In our study, we constructed a signature consisting of 12 VRGs which was prognostic biomarker of HCC, including CYP24A1, NCOA7, TGFB1, GSR, IGFBP3, IGFBP2, VGF, AGAP2, DENND6B, LRRC8A, BCL6, FCER2. TGFB1 was involved in the aggravation of HCC malignant behaviors.^[16]^ IGFBP3 inhibit cell growth and insulin-like growth factor signaling in HCC.^[17]^ LRRC8A served as a central mediator and accelerated colon cancer metastasis by regulating PIP5K1B/PIP2 pathway.^[18]^ NCOA7 could regulates growth and metastasis of clear cell renal cell carcinoma via MAPK/ERK Signaling Pathway.^[19]^ IGFBP3 promotes resistance to Olaparib via modulating EGFR signaling in advanced prostate cancer.^[20]^

One of the best therapy options for the patients that could not receive operation is immunotherapy.^[21]^ However, there are limited sensitive markers for the prediction of immunotherapy benefits though many immune checkpoint-based drugs have been used clinically. In our study, the value of VRS in immunotherapy benefit prediction of patients. There is a higher immune escape likelihood and less immunotherapy benefits in patients with higher TIDE score.^[22]^ Higher TMB score suggested betterimmunotherapy benefit.^[23]^ We found a higher TMB score and lower TIDE score and tumor escape score in HCC patients with low-risk score. Immunophenotype score, primarily developed from TCGA RNA-seq data, was designed to predict patient responses to immune checkpoint inhibitor treatments.^[24]^ It is confirmed that low-risk populations respond more effectively to immunotherapy, as evidenced by the higher immunophenotype score, which was consistent with our findings. Thus, low VRS-based risk score may indicate a batter response to immunotherapy.

We then performed gene set enrichment analysis to explore the biological process in HCC patients with different VRS score. The result suggested HCC patients with high-risk score had a higher score of angiogenesis, coagulation, glycolysis, hypoxia, MTORC1 signaling, and P53 pathway, indicating that the activation of these biological processes may play the vital role in HCC tumorigenesis and progression. Angiogenesis played a vital role in the developed HCC.^[25]^ Hypoxia was also involved in innate immunity and tumor progression in HCC.^[26]^ Glycolysis shows significant correlation with the prognosis of HCC.^[27]^

## 5. Conclusion

Our study proposed a novel VRS for HCC, which served as an indicator for predicting clinical outcome and immunotherapy response in HCC.

## Author contributions

**Data curation:** Bin Guo.

**Formal analysis:** Lulu Han.

**Investigation:** Tianyi Wang.

**Methodology:** Lulu Han, Jinjiang Xu.

**Project administration:** Bin Guo.

**Software:** Jinjiang Xu.

**Validation:** Lulu Han.

**Writing – original draft:** Tianyi Wang.

**Writing – review & editing:** Jinjiang Xu, Bin Guo.

## Supplementary Material


